# Evaluation of diagnostic ultrasound use in a breast cancer detection strategy in Northern Peru

**DOI:** 10.1371/journal.pone.0252902

**Published:** 2021-06-11

**Authors:** Segen Aklilu, Carolyn Bain, Pooja Bansil, Silvia de Sanjose, Jorge A. Dunstan, Vanesa Castillo, Vivien Tsu, Ines Contreras, Ronald Balassanian, Tara K. Hayes Constant, John R. Scheel

**Affiliations:** 1 Department of Radiology, University of Washington, Seattle, Washington, United States of America; 2 PATH, Seattle, Washington, United States of America; 3 Instituto Nacional de Enfermedades Neoplásicas, Lima, Peru; 4 PATH, Lima, Peru; 5 Department of Global Health, University of Washington, Seattle, Washington, United States of America; 6 Department of Pathology, University of California San Francisco, San Francisco, California, United States of America; 7 Sea Mar Community Health Centers, Burien, Washington, United States of America; Brown University Warren Alpert Medical School, UNITED STATES

## Abstract

To evaluate the diagnostic impact of point-of-care breast ultrasound by trained primary care physicians (PCPs) as part of a breast cancer detection program using clinical breast exam in an underserved region of Peru. Medical records and breast ultrasound images of symptomatic women presenting to the Breast Cancer Detection Model (BCDM) in Trujillo, Peru were collected from 2017–2018. Performance was measured against final outcomes derived from regional cancer center medical records, fine needle aspiration results, patient follow-up (sensitivity, specificity, positive, and negative predictive values), and by percent agreement with the retrospective, blinded interpretation of images by a fellowship-trained breast radiologist, and a Peruvian breast surgeon. The diagnostic impact of ultrasound, compared to clinical breast exam (CBE), was calculated for actual practice and for potential impact of two alternative reporting systems. Of the 171 women presenting for breast ultrasound, 23 had breast cancer (13.5%). Breast ultrasound used as a triage test (current practice) detected all cancer cases (including four cancers missed on confirmatory CBE). PCPs showed strong agreement with radiologist and surgeon readings regarding the final management of masses (85.4% and 80.4%, respectively). While the triage system yielded a similar number of biopsies as CBE alone, using the condensed and full BI-RADS systems would have reduced biopsies by 60% while identifying 87% of cancers immediately and deferring 13% to six-month follow-up. Point-of-care ultrasound performed by trained PCPs improves diagnostic accuracy for managing symptomatic women over CBE alone and enhances access. Greater use of BI-RADS to guide management would reduce the diagnostic burden substantially.

## Introduction

The low awareness and lack of local access to medical imaging result in most women with breast cancer in low- and middle-income countries (LMICs) presenting at a late stage when treatment is largely unsuccessful [1–5]. A multi-organization consortium, including PATH, the Peruvian Ministry of Health, and the national and regional cancer centers (INEN and IREN-Norte), the University of Washington, and the University of California San Francisco, instituted the Breast Cancer Detection Model (BCDM) in Northern Peru using locally available resources [6, 7]. Peru was chosen because of the government-run Seguro Integral de Salud, which offers free cancer care in-country, and Plan Esperanza initiative which aids in educational activities. The BCDM focuses on improved access to accurate clinical breast exams (CBEs) in local communities by raising breast health awareness among women and providing education and training to midwives and primary care physicians (PCPs).

WHO defines early diagnosis of cancer as focusing on detecting symptomatic patients as early as possible, so they have the best chance for successful treatment (https://www.who.int/activities/promoting-cancer-early-diagnosis). This contrasts to screening, which occurs in asymptomatic women. Screening is a more advanced level of care delivery that requires more resources and must be preceded by an established early diagnosis program.

The BCDM also uses implementation science principles by introducing evidence-based interventions into a different clinical setting and evaluating their impact. Interventions include training to build skills and induce behavioral changes among health care providers. The overall aim is the adoption of improved CBE and ultrasound imaging, education to engage patients and stakeholder organizations, and revised management algorithms [8].

Previous research has shown that breast ultrasound is effective in LMICs to detect breast cancer, particularly in conjunction with CBE [9, 10]. Ultrasound use has reduced the rate of abnormal CBEs requiring tissue sampling by up to 75% [11]. Ultrasound also provides the PCP and patient with reassurance by identifying benign (e.g., cysts) or normal (e.g., ribs) findings that correspond to the abnormal CBE. Ultrasound was introduced into the BCDM patient care pathway as a low-cost approach to increase access at the point-of-care in the community and potentially reduce the number of women requiring fine needle aspiration (FNA) at more centralized health centers.

In the U.S., radiologists report ultrasound findings using the Breast Imaging Reporting and Data System (BI-RADS) [12]. Despite the wealth of data supporting BI-RADS use to improve interpretive accuracy, it is not clear that PCPs and technologists interpreting ultrasounds can use the full BI-RADS accurately to inform management of breast findings. Previous studies indicate that, among LMIC radiologists not fully trained using BI-RADS, use of BI-RADS can result in mis-characterization and mis-management of findings. However, standardized systems using fewer terms and decision points appear to result in more consistent and accurate management [7].

Program evaluations on breast imaging interpretation can improve accuracy and efficiency by addressing interobserver variability and providing practitioners with feedback [7, 10, 13, 14]. For example, variability in lesion margin description, a critical factor in the decision for biopsy, may affect biopsy rates and missed cancer rates [15]. Among U.S. radiologists, there are variable interobserver rates with fair agreement with respect to descriptors for lesion echo pattern or margin [16, 17], as well as fair or poor agreement in assigning some assessment categories [15, 18].

The purpose of the current assessment is to evaluate the ultrasound component of the BCDM, regarding the accuracy of breast ultrasound performance and interpretation by trained PCPs in the La Libertad region, and evaluate the diagnostic impact of adding ultrasound to standard CBE. A secondary goal of this program evaluation is to assess the theoretical diagnostic impact of different reporting systems (triage, condensed BI-RADS, full BI-RADS). The results of this evaluation will provide data for the potential scalability of this model in other regions of Peru and help improve the program during the scale-up phase.

## Methods

### Ethics statement

This retrospective study was exempt from IRB review by the University of Washington and PATH due to its retrospective design and use of anonymized data. The original program was exempted from IRB review by the PATH IRB committee as the program provided standard of care to a region currently receiving no early diagnosis activities. This program was not intended for research and, therefore, women were not consented.

### Study setting

La Libertad is a medically underserved region in northern Peru, 560 km north of the more resourced capital city of Lima. Trujillo is the largest city in La Libertad; here women have access to basic primary care services, but not routine mammography screening.

As part of BCDM, trained clinic-based midwives educated women on breast cancer and performed CBEs. Women with signs and symptoms of potential breast cancer (e.g., breast lump) in Trujillo were referred to a PCP for a CBE, ultrasound and an FNA. Clinic staff transport FNA samples to IREN-Norte for interpretation by a cytopathologist.

### Patient and public involvement

There was a collaborative effort from the national government, the Peruvian Ministry of Health and other local and foreign health agencies. These entities were involved in the clinical algorithm/management, recruitment and conduct of the overall program.

### Intervention

For this study, symptomatic women were defined as those presenting with either a self-detected symptom or a positive clinical finding by the midwife (e.g., lump, skin changes). The BCDM incorporated breast ultrasound in 2016; a trained PCP performed and interpreted the breast ultrasound after their CBE and prior to the decision regarding FNA. If either the ultrasound or the PCP CBE was positive, FNA was performed immediately. The addition of ultrasound to the program consisted of ultrasound training of the PCPs, quality assurance, a revised management algorithm, and data collection (S1 Fig).

#### Ultrasound training

A Peruvian breast surgeon from INEN with previous breast ultrasound experience trained for two weeks with a fellowship-trained breast radiologist at Seattle Cancer Care Alliance/University of Washington. Thereafter, the surgeon trained 15 PCPs (in two groups) in the La Libertad region. The training lasted eight hours a day for two days and included both didactic and practical components. After the training, a trained PCP from the first group mentored PCPs in the second group. This second group received oversight and BI-RADS training from the surgeon and were required to pass a written test before practicing independently. A similar multi-tiered training approach has been used in other countries [7].

#### Quality assurance

The breast surgeon reviewed ultrasound images to assure quality. Patients with a negative or benign interpretation by the PCPs subsequently interpreted as positive by the surgeon were contacted and received appropriate care. Interpretive performance (i.e., medical audit) was calculated using the BI-RADS Atlas guidelines [12].

#### Development of an algorithm for evaluating patients with ultrasound

A patient management algorithm (S2 Fig) was developed to standardize decision-making. To reduce the possibility of missing a cancer, the algorithm calls for ultrasound to be used as a triage tool to differentiate normal from abnormal breast tissue, with immediate FNA for all findings (BI-RADS 3, 4, and 5) except normal breast tissue and classically benign findings (BI-RADS 1 and 2).

### Imaging

All ultrasound imaging was performed with high frequency linear transducers (>7.5 MHz) on either a VINNO E30 (Sozhou, China) or a SAMSUNG MEDISON ACUVIX XG (Seoul, Korea). Physicians recorded their ultrasound interpretation on standardized forms. Such forms have been shown previously to improve interpretation and management of findings by PCPs and facilitate learning of BI-RADS [7].

### Data collection

In addition to clinical intake forms and ultrasound images, BCDM program staff collected medical records and patient follow-up details from IREN-Norte (cancer versus no cancer). Positive outcomes were defined as ultrasound examinations associated with a cancer diagnosis within 365 days. Ultrasound examinations not associated with a cancer diagnosis by FNA or after searching the IREN-Norte medical records after 365 days from ultrasound were considered negative [12]. The breast radiologist (7 years experience) and breast surgeon (1 year experience) retrospectively reviewed and interpreted anonymized ultrasound images, blinded to each others’ interpretations and the initial interpretations by the PCPs. Both reviewers completed the same ultrasound interpretation form used by the PCPs.

### Statistical analysis

All statistical analyses are conducted in Stata 13.1 (StataCorp, College Station, TX). The distribution of mass characteristics (shape, internal echogenicity, margins, and size) were used to calculate the likelihood of malignancy [19]. Cancer detection rate (per 1,000 ultrasounds), abnormal interpretation rate, sensitivity, specificity, positive predictive values for ultrasound-positive women recommended for biopsy and for women actually biopsied (PPV2, PPV3), and negative predictive value (NPV), were calculated according to the 5th edition BI-RADS Atlas [12]. Performance was also calculated in terms of percent agreement between interpreting groups (radiologist and surgeon, surgeon and PCPs, and radiologist and PCPs) for the BI-RADS lexicon, findings, and management plans. The patient management algorithm recommended biopsy for all masses (Triage ultrasound), even those with probably benign findings. Therefore, BI-RADS 3, 4, and 5 findings were considered a positive interpretation. In contrast, when calculating the theoretical interpretive performance of using condensed or full BI-RADS system, masses with probably benign characteristics (BI-RADS 3) would be followed by repeat imaging six months later and, therefore, considered a negative interpretation in accordance with the BI-RADS Atlas [12].

The diagnostic impact of breast ultrasound was calculated by assuming that, in the absence of breast ultrasound, every suspicious CBE finding receives a standard-of-care referral for a surgical biopsy. Management changes after breast ultrasound, according to actual practice (i.e., triage) and potential practice (condensed or full BI-RADS), were determined.

## Results

### Patient population

**Fig 1:**
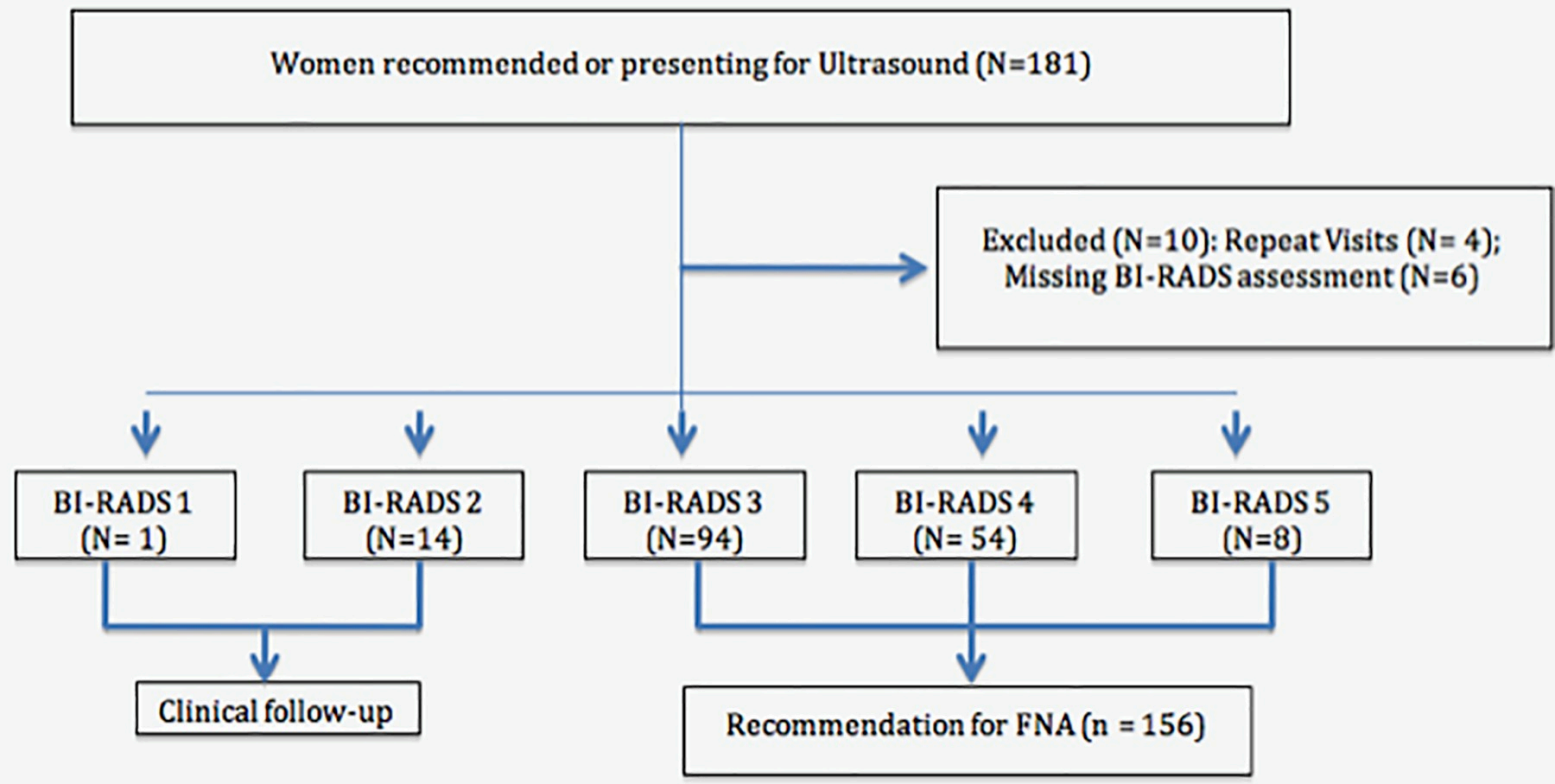
Flowchart for ultrasound examination results.

From 2017–2018, 181 diagnostic ultrasounds were performed on symptomatic women referred to the BCDM PCP for evaluation (Fig 1). Ten (5.5%) were excluded: four repeat visits of women already included in the analysis and six missing a BI-RADS assessment. Out of 171, 156 (91.2%) were recommended for FNA (BI-RADS 3–5), 103 (66%) received an FNA within the program and 48 (30.8%) received appropriate follow-up outside the program. Five women (3.2%) recommended for FNA did not have FNA or appropriate follow-up documented. Twenty-three cancers were diagnosed during the study period.

S1 Table describes the distribution of characteristics for the 171 patients included in this program evaluation. Of the 171 women, 56% were 30–49 years old and 69% breast-fed their children. Many reported contraception use (40.1%), while few reported alcohol use (1.7%). Few women had family history (4.5%) and none had personal history of breast cancer.

### Interpretive performance of PCPs

Table 1 shows the frequency of characteristics used by PCPs to describe masses, with associated likelihood of malignancy and benignity. Masses with oval/round shape (n = 83) were more common than irregular shape (n = 69) and were significantly less likely to represent malignancy (7.2% versus 24.6%, p = < 0.001). Similarly, circumscribed margins (n = 92) were more common than not circumscribed margins (n = 60) and were significantly less likely to be associated with malignancy (9.8% versus 23.3%, p = 0.049). All masses associated with cancer (n = 23) were assigned not hyperechoic for internal echogenicity. Larger masses were more likely to represent malignancy (p = 0.01).

**Table 1:** Characteristics used to describe masses on ultrasound (N = 156).

Ultrasound Mass Characteristics	Number (%)	Number (%) with cancer diagnosis	Number (%) without cancer diagnosis	P value*
Shape				
Irregular	69(44.2)	17 (24.6)	52 (75.4)	<0.001
Oval/round	83(53.2)	6 (7.2)	77 (92.8)	
Missing	4(2.6)	0 (0)	4 (100.0)	
Internal echogenicity				
Hyperechoic	2(1.2)	0 (0)	2 (100.0)	0.70
Not Hyperechoic	152(97.4)	23 (15.1)	129 (84.9)	
Missing	2(1.2)	0 (0)	2 (100.0)	
Margins				
Circumscribed	92(58.9)	9 (9.8)	83 (90.2)	0.049
Not Circumscribed	60(38.5)	14 (23.3)	46 (76.7)	
Missing	4(2.6)	0 (0)	4 (100.0)	
Size				
Size > 2 cm	58(37.2)	15 (25.9)	43 (74.1)	0.010
Size (2 cm or less)	91(58.3)	7 (7.7)	84 (92.3)	
Missing	7(4.5)	1 (14.3)	6 (85.7)	
Total	156 (100)			

*Chi-squared P value

There was strong agreement between all groups for identifying the correct finding (88–89%) (Table 2). The percent agreement for all BI-RADS lexicon characteristics for masses was highest for internal echogenicity (97–98%), followed by margins (60–86%), and shape (70–84%). Percent agreement between all groups for the final assessment of masses was highest using triage (80–85%), compared to full BI-RADS (47–54%) or condensed BI-RADS (50–56%).

**Table 2:** Inter-observer percent agreement for BI-RADS lexicon use and assessment.

	BR vs. S	BR vs. PCPs	S vs. PCPs
Overall Findings	88.5	88.4	88.3
For masses			
Number of masses	137	146	143
Type of Shape	70.3	83.9	71.5
Type of Margin	85.8	64.2	59.5
Internal Echogenicity	97.7	97.1	98.4
Assessment			
Number of women assessed	156	164	153
Full BI-RADS	53.8	48.8	47.1
Condensed BI-RADS§	56.4	54.9	50.3
Triage Ultrasound¶	79.5	85.4	80.4

BR: breast radiologist; S: Peruvian Breast Surgeon; PCPs: primary care physicians

§ Condensed BI-RADS: BI-RADS 1 and 2, BI-RADS 3, BI-RADS 4 and 5

¶ Triage Ultrasound: BI-RADS 1 and 2; BI-RADS 3 and 4 and 5

Triage ultrasound (BR 3,4,5 considered positive) identified four cancers considered not suspicious by the PCP—two called negative and two assigned for clinical follow-up (Table 3). Triage ultrasound was more sensitive than the PCP’s CBE (100% and 82.6% respectively). Furthermore, using ultrasound as a triage test also resulted in a somewhat higher sensitivity (100% versus 87%) and NPV (100%) than condensed and full BI-RADS, but with significantly lower specificity (10.1% versus 71.6%) (Table 4). The condensed and full BI-RADS would have resulted in 94 BI-RADS 3 (probably benign) masses being followed-up with repeat imaging later, rather than immediate biopsy, with 3.2% of them ending up being malignant (S2 Table).

**Table 3:** Clinical management changes before and after breast ultrasound for all women.

	Physicians’ CBE management		Post-CBE Ultrasound Management			
			Management Category	Triage System	Condensed System	Cancers detected
All women (N = 171)	Negative	26 (15.2)	Imaging follow-up	0	14 (53.9)	0
			Biopsy	25 (96.1)	11 (42.3)	2
			Negative/Benign	1 (3.9)	1 (3.9)	0
	Follow up	14 (8.2)	Imaging follow-up	0	8 (57.1)	0
			Biopsy	11 (78.6)	3 (21.4)	2
			Negative/Benign	3 (21.4)	3 (21.4)	0
	Referral with biopsy	131 (76.6)	Imaging follow-up	0	72 (55.0)	3
			Biopsy	120 (91.6)	48 (36.6)	16
			Negative/Benign	11 (8.4)	11(8.4)	0
No. (%) Biopsies by Recommendation		131 (76.6)		156 (91.2)	62 (36.2)	23 (100)
False Positive Rate		85.4		85.3	67.7	

**Table 4:** Interpretive performance of primary care physicians based on actual and alternative reporting systems (N = 171).

	Triage Ultrasound†	Condensed/Full BI-RADS‡
Cancer Detection Rate (per 1,000 ultrasounds)	147.4	322.6
Abnormal interpretation rate	88.1	35.0
Sensitivity	100.0 (85.2–100.0)	87.0 (66.4–97.2)
Specificity	10.1 (5.8–16.2)	71.6 (63.6–78.7)
PPV2	N/A	32.3 (20.9–45.3)
PPV3	N/A	45.5 (30.4–61.2)
NPV	100.0 (78.2–100.0)	97.2 (92.2–99.4)

†Triage ultrasound: BI-RADS 3,4,5 are positive interpretations and result in a biopsy recommendation

‡Condensed and full BI-RADS: BI-RADS 4,5 are positive interpretations and result in a biopsy recommendation

PPV2: BI-RADS 4 and 5 as positive interpretation; women recommended for biopsy with missing FNA results are assumed negative

PPV3: BI-RADS 4 and 5 as positive interpretation; women recommended for biopsy with missing FNA results are excluded

### Diagnostic impact of ultrasound on the management of symptomatic women

Based on the PCP’s CBE alone, 76.6% of the women would have been referred for ultrasound, while 8.2% and 15.2% would be designated as clinical follow-up (non-neoplastic abnormality) and negative (no follow-up needed) respectively (Table 3). In total, ultrasound changed the management after the PCP’s CBE in 29.2% of cases with triage (mostly increasing biopsies), and 66.7% with condensed and full BI-RADS (mostly decreasing biopsies). The false positive rate for biopsy recommendations was 85.4% for the PCP’s CBE alone, 85.3% for ultrasound triage, and 67.7% for condensed BI-RADS.

## Discussion

Evaluating programs is a critical component of implementation science and involves quality assessment, improvement, and sustainability. Here, the impact of imaging was evaluated in the BCDM in an underserved region in Peru. This is one of the first studies–outside clinical trials–following the use of CBE, education, and point-of-care ultrasound that included multiple years of program follow-up on patient outcomes and several generations of PCPs, trained by in-country trainers, performing ultrasound. The results suggest that PCP’s CBE followed by point-of-care triage ultrasound and tissue sampling with FNA performed by PCPs can increase clinical accuracy at the primary care level, reducing consumption of limited specialty care services in resource-limited settings. Further, different standardized reporting systems for ultrasound demonstrated important trade-offs to consider when starting similar programs.

Several groups have used education with point-of-care CBE and ultrasound at community health centers and through periodic camps to decentralize diagnostic services and increase access [7, 11, 20]. These efforts have limited sustainability without continued external resources or commitment from the government. By training a local breast surgeon in breast ultrasound who could train and support subsequent generations of PCPs, using pre-existing imaging equipment, and shifting ultrasound interpretation and FNA to PCPs, the program becomes more sustainable with local resources. The ability of PCPs to achieve improved results with ultrasound suggests this approach is viable. Although the level of agreement was reduced significantly when using more detailed BIRAD reporting systems (condensed and full BI-RADS), the disagreement was usually caused by ‘overcalling’ benign findings as probably benign, and probably benign as suspicious. This cautious approach among less experienced radiologists has been observed in the U.S. and normalizes with experience and feedback [21]. Further follow-up is necessary to confirm that PCP patient management agreement improves as experience increases, provided that examinations are linked to patient follow-up information. Ensuring continued improvement in image interpretation through increased training and feedback is necessary to maximize the impact of ultrasound.

Most diagnostic services in LMICs are centrally located in large cities, and women with late- stage breast cancer often cite distance from these tertiary diagnostic centers and limited knowledge about their locations as factors that contributed to delayed presentation [22]. In northern Peru, symptomatic women required further evaluation at distant tertiary hospitals. In our study, 89% received ultrasound and FNA by a trained PCP in the community close to where the women live. These rates were better than a similar program in rural Uganda (36%) that offered point-of-care ultrasound locally, but FNA at a tertiary hospital farther away [11]. These findings suggest that breast cancer detection services, linked to pathology services, closer to where women live can decrease the women’s burden and increase successful detection and follow-up. Importantly, reducing false positive evaluations and unnecessary biopsies saves women money, time, and anxiety, and may improve community participation in a breast cancer detection program over time. In this evaluation, point-of-care ultrasound performed by PCPs increased biopsies by 14.6% using the conservative triage algorithm (while improving sensitivity over PCP CBE), but could have reduced biopsies by 40.4% if the condensed BI-RADS had been used. Prior studies in High Income Countries and LMICs have shown ultrasound improves the sensitivity and specificity of CBE [9, 10, 23]. One study in Uganda showed ultrasound reduced false positive CBEs requiring tissue sampling by 75%. However, the median age of women in that study was 29 years–ten years younger than our study [11].

There are clear trade-offs between the simplicity of triage ultrasound and more complex condensed or full BI-RADS approaches. While the triage system was easier to learn and use consistently, it resulted in greater resource utilization by recommending biopsy of all masses (BI-RADS 3, 4, and 5); this is expensive and potentially unnecessary in LMICs where most women presenting for an evaluation (early adopters) are < 40 years old and thus relatively low risk for breast cancer. However, the recommendation to use imaging follow-up to manage probably benign masses (BI-RADS 3) instead of immediate biopsy relies on an assumption of <2% risk of malignancy [12] and an assurance that women have access to subsequent imaging follow-up. Using condensed BI-RADS would have resulted in short interval follow-up of three masses that represented cancer (3.2%). Although this is above the cutoff level recommended in the U.S. [12], health systems will need to consider the tradeoff with the number of women who might not receive care due to the added burden of biopsying numerous benign masses. Each reporting system (triage, condensed BI-RADS, full BI-RADS) varies in complexity. One approach is to start with the simplest reporting system (triage) and gradually phase out FNA of “probably benign” findings, as quality assurance reviews provide evidence that it is safe to manage such findings without immediate FNA, as suggested by Lam et al. [24]. Program planners should initially anticipate providers needing greater support and oversight, gradually shifting towards a more comprehensive reporting system that allows imaging follow-up when the infrastructure and diagnostic performance are ready.

It is notable that one third of the cancers detected by the BCDM program were 2 cm or less. This observation is consistent with those from large randomized controlled trials in China and India, where approximately 45% and 19% of masses detected by a combination of awareness, self-detection, and CBE were less than 2 cm, respectively [25, 26]. These results support the argument that even in settings without mammography, breast cancer early diagnosis is feasible.

This study has limitations. While using BI-RADS 3 imaging follow-up, as an alternative to sampling all masses, will save money, data was not collected to calculate cost-effectiveness of this option and determine how many more women would receive care over use of triage ultrasound. Additionally, it is still possible, although unlikely given limited healthcare options in this region, that some women receive follow-up outside our program [6]. Patient follow-up information was obtained through the cancer center and repeat community visits, where possible, to reduce the likelihood of missed cancers and patients lost to follow-up. Searching records of local facilities found follow-up for eight additional women without FNA or follow-up results at our study facility. Lastly, the study results have potentially limited generalizability to older Peruvian women because only 45% of women included in this study were 40 years or older; however, all cancers detected were in this older group. Including a more significant proportion of women at higher risk for malignancy could likely show improved efficiency of cancer detection of our strategy. Nevertheless, although important, these limitations do not inhibit our ability to improve cancer detection in this region.

## Conclusion

Adding point-of-care ultrasound with FNA to local breast education and CBE services may improve early breast cancer diagnosis and can potentially reduce unnecessary biopsies using more advanced ultrasound reporting systems. This strategy saves resources and improves early cancer detection over existing options in LMICs. Early diagnosis breast cancer programs should start with a basic standardized reporting system for ultrasound. Once infrastructure and experience are in place, it is possible to transition to more comprehensive and selective approaches to save resources.

## Supporting information

S1 Fig Translated clinical intake form for breast ultrasound. (PDF)

S2 Fig Flowchart of services in community program for breast health. (PDF)

S1 Table Population characteristics of symptomatic women presenting for a breast evaluation (N = 171). (PDF)

S2 Table Accuracy of PCP assessment in detecting cancers, by actual and potential reporting system (N = 171). (PDF)
