# Supplementary material for: Evaluation of diagnostic ultrasound use in a breast cancer detection strategy in Northern Peru
Source: PLoS One. 2021 Jun 11;16(6):e0252902. doi: 10.1371/journal.pone.0252902 (PMC8195385; doi:10.1371/journal.pone.0252902)
Supplement: S1 Fig — (PDF) [file pone.0252902.s001.pdf]

S1 Fig. Translated clinical intake form for breast ultrasound

<table>
<tr><td>SUBJECTIVE
(Informed by the patient)</td><td colspan="2">Patient Characteristics:
Name of patient ________________________
ID____________________________ Age_____
Main symptom___________________________
Beginning of symptom
(MM/YYYY)_______/___________
Number of tumors*: ______________________
*Fill out one format for each mass
Signed Informed Consent: □ YES □ NO
Is the patient referred at the presence of a breast abnormality?
□ YES □ NO

Does the patient state the mass is not painful?
□ YES □ NO
Does the patient state any change in the shape or size of the breast?
□ YES □ NO
Does the patient state having any personal or family history of breast or ovarian cancer?
□ YES □ NO
Does the patient state having nipple secretion?
□ YES □ NO</td></tr>
<tr><td></td><td>1) Clinical Breast Exam (CBE)
Date (DD/MM/YYYY): ______/______/______</td><td>2) Breast ultrasound
Date (DD/MM/YYYY): ______/______/______</td></tr>
<tr><td>OBJECTIVES
(Observed by the physician)</td><td>Consistency: □ Soft □ Hard
Mobility: □ Mobile □ Fixed
Borders: □ Exact □ Without definition
Surface: □ Regular □ Irregular
Pain: □ YES □ NO
Bilaterally: □YES □ NO

Skin:
□ Nipple secretion □ “Orange peel skin”
□ Cutaneous erythema □ Skin retraction
□ Changes in nipple direction
□ Increase in surface vein network

Lymph nodes: □ Clavicular area □ Axillary

Location:
□ Left □ Right □ Axilla □ Sub-areola
Distance from nipple _________ cm
Clockwise position ________
Diameter _________ cm</td><td>Margin: □ Delimited □ Not delimited

Shape: □ Oval/Round □ Irregular

Echogenicity (in relation to fat):
□ Hypo-echoic/Iso-echoic
□ Anechoic
□ Hyper – echoic
□ Complex echo texture

Diameter: _______ cm

BIRADS:
□ 0 Incomplete, needs additional imaging
□ 1 Negative
□ 2 Benign Finding(s)
□ 3 Probably benign
□ 4 Suspicious of abnormality
□ 5 High likelihood of neoplasm</td></tr>
<tr><td>EVALUATION</td><td>Clinical Breast Exam Finding:
□ Normal (no mass) □ Suspicious of cancer
□ Seems benign □ High suspicious of cancer

Interpretation:
□ Without focal findings in CBE
□ Focal findings in CBE</td><td>Breast Ultrasound Finding:
□ Normal Tissue □ Mass □ Cyst □ Infection □ Lymph node □ Fat necrosis
□ Galactocele
Interpretation:
□ Suspicious of cancer in ultrasound
□ Not suspicious of cancer in ultrasound:
□Cyst
□ Cyst + solid component
□ Benign mass</td></tr>
<tr><td>PLAN</td><td>Clinical plan previous to any ultrasound:
□ Control or Follow up:
□ 3 months □ 6 months □ 12 months (1 yeas)
□ Recommendation for a local ultrasound
□ Direct referral to an Oncologic Hospital and look for counter referral in 1 month</td><td>Clinical Plan after ultrasound:
□ Clinical Management:
□ Control ____ months
□ Cyst drainage:
Bloody? □ YES □ NO
□ Taking of sample FNA mass
□ Direct referral to an Oncologic Hospital and look for counter referral in 1 month</td></tr>
<tr><td>NOTES</td><td colspan="2">Other observations of the physician:

With cytological sample (FNA):
Date of cytopathologist reception (DD/MM/YYYY): ______/______/______
Cytology Result: □ Negative for malignant neoplasm □ Cytology of carcinoma
□ Atypical cytology □ Unsuitable sample</td></tr>
</table>
