# Supplementary material for: Evaluation of diagnostic ultrasound use in a breast cancer detection strategy in Northern Peru
Source: PLoS One. 2021 Jun 11;16(6):e0252902. doi: 10.1371/journal.pone.0252902 (PMC8195385; doi:10.1371/journal.pone.0252902)
Supplement: S2 Fig — (PDF) [file pone.0252902.s002.pdf]

S2 Fig. Flowchart of services in community program for breast health

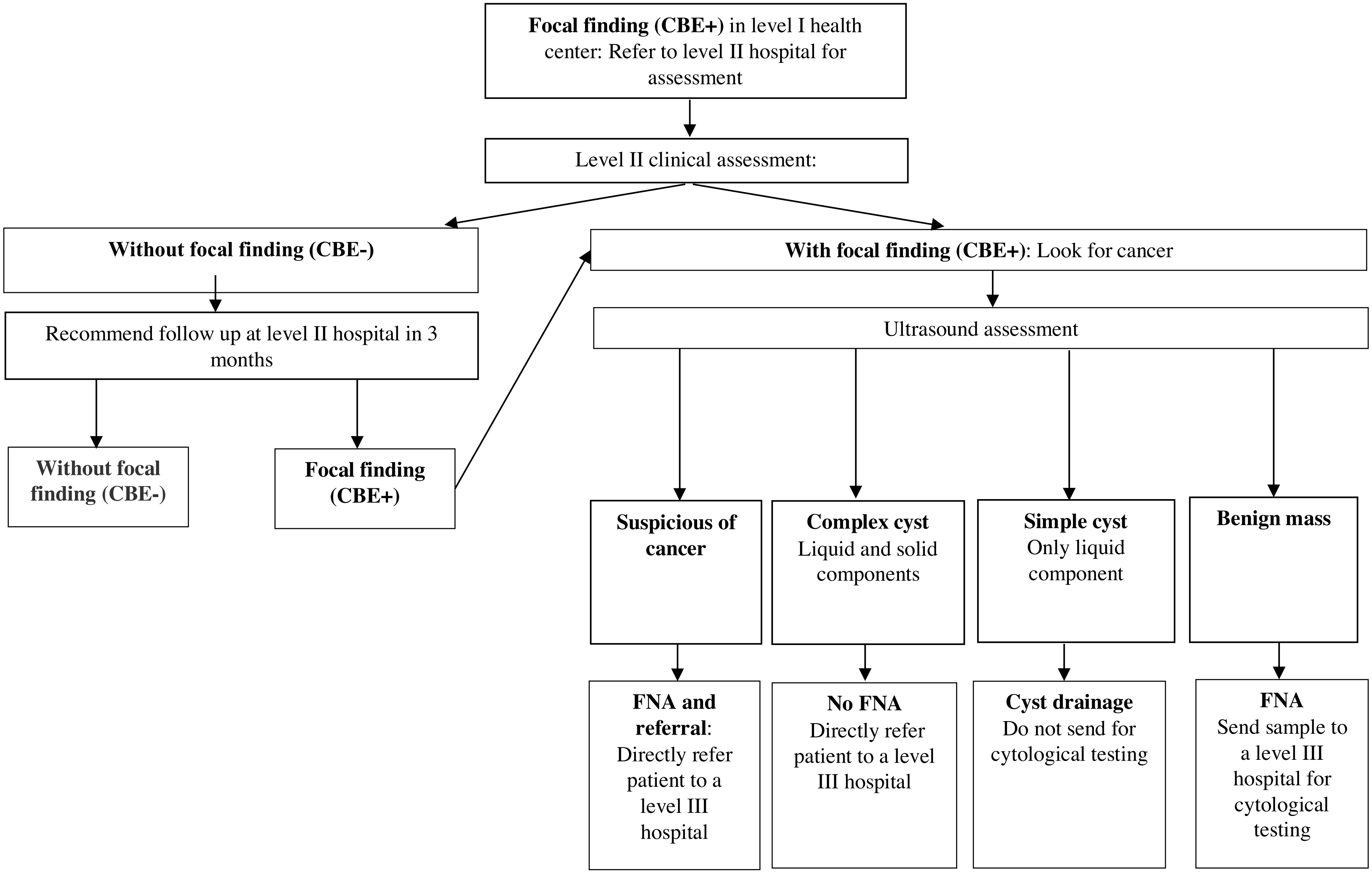

See **page 2** of flowchart – clinical question based on cytological findings

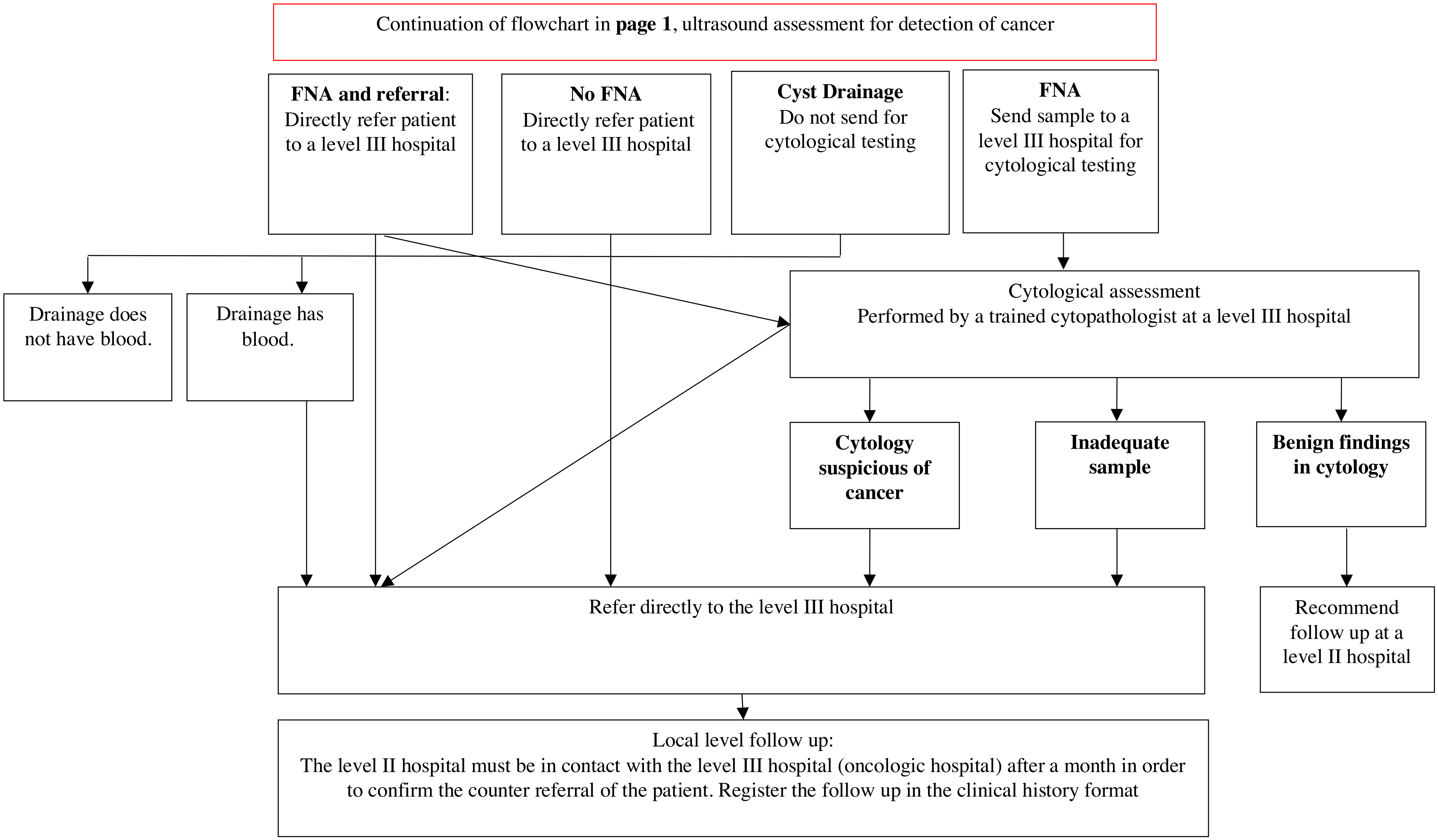
Continuation of flowchart in page 1, ultrasound assessment for detection of cancer
FNA and referral: Directly refer patient to a level III hospital
No FNA Directly refer patient to a level III hospital
Cyst Drainage Do not send for cytological testing
FNA Send sample to a level III hospital for cytological testing
Drainage does not have blood.
Drainage has blood.
Cytological assessment Performed by a trained cytopathologist at a level III hospital
Cytology suspicious of cancer
Inadequate sample
Benign findings in cytology
Refer directly to the level III hospital
Recommend follow up at a level II hospital
Local level follow up: The level II hospital must be in contact with the level III hospital (oncologic hospital) after a month in order to confirm the counter referral of the patient. Register the follow up in the clinical history format
