# Supplementary material for: Evaluation of diagnostic ultrasound use in a breast cancer detection strategy in Northern Peru
Source: PLoS One. 2021 Jun 11;16(6):e0252902. doi: 10.1371/journal.pone.0252902 (PMC8195385; doi:10.1371/journal.pone.0252902)
Supplement: S1 Table — (PDF) [file pone.0252902.s003.pdf]

S1 Table. Population characteristics of symptomatic women presenting for a breast evaluation (N=171)

Characteristics	***N (%)***
Age	
<20	13 (7.3)
20–29	32 (18.1)
30–39	52 (29.4)
40–49	47 (26.6)
≥50	33 (18.6)
Median (range)	39 (14 – 80)
Breast Cancer Risk and protective Factors	
Family history of Breast CA (yes)	8 (4.5)
Family history of Ovarian CA (yes)	4 (2.3)
Personal history of Breast CA (yes)	0 (0)
Contraception Use (yes)	71 (40.1)
Hormone therapy use (yes)	1 (0.6)
Alcohol (yes)	3 (1.7)
Breast fed (yes)	122 (68.9)
Age of Menarche (≤11)	38 (21.5)
Age of first pregnancy (≥30 years)	16 (9.0)
Age at menopause (≥54)	1 (0.6)
