# Supplementary material for: Evaluation of diagnostic ultrasound use in a breast cancer detection strategy in Northern Peru
Source: PLoS One. 2021 Jun 11;16(6):e0252902. doi: 10.1371/journal.pone.0252902 (PMC8195385; doi:10.1371/journal.pone.0252902)
Supplement: S2 Table — (PDF) [file pone.0252902.s004.pdf]

S2 Table. Accuracy of PCP assessment in detecting cancers, by actual and potential reporting system (N= 171)

BI-RADS Category	No. of women by Triage (% Cancers detected)	No. of women by Condensed BI-RADS (% Cancers detected)	No. of women by Full BI-RADS (% Cancers detected)	No. of Cancers Detected
1	15 (0)	15 (0)	1 (0)	0
2			14 (0)	0
3	156 (14.7)	94 (3.2)	94 (3.2)	3
4		62 (32.3)	54 (27.8)	15
5			8 (62.5)	5
Total	171 (13.5)	171 (13.5)	171 (13.5)	23
